# Pore-Scale Investigations into Gradient Carbon Microstructures for Enhanced Mass Transport in PEM Fuel Cell Catalyst Layers

**DOI:** 10.3390/nano16020088

**Published:** 2026-01-09

**Authors:** Chao Zhang, Lingquan Li, Hao Wang, Guogang Yang, Naibao Huang, Zhonghua Sheng

**Affiliations:** 1 Marine Engineering College, Dalian Maritime University, Dalian 116026, China; zhangchao0123@dlmu.edu.cn (C.Z.); li_0203@dlmu.edu.cn (L.L.); nbhuang@dlmu.edu.cn (N.H.); 2 College of Materials and Metallurgy, University of Science and Technology Liaoning, Anshan 114051, China; zhhsheng1993@163.com

**Keywords:** catalyst layer microstructure, carbon sphere diameter distribution, proton exchange membrane fuel cells, lattice Boltzmann method, oxygen transport and electrochemical performance

## Abstract

This study investigates the impact of non-uniform carbon sphere diameter distributions on the structural and electrochemical performance of catalyst layers (CLs) in proton exchange membrane fuel cells (PEMFCs), utilizing the lattice Boltzmann method (LBM) for detailed simulations. The impact of carbon sphere diameter range and gradient distribution on oxygen transport, electrochemical reactivity, and catalyst layer morphology was investigated. The results show that gradient designs of carbon sphere diameters effectively modulate pore size distribution, electrochemically active surface area, and oxygen diffusion pathways within the CL. Specifically, placing larger carbon spheres near the gas diffusion layer improves pore connectivity and oxygen transport, while smaller spheres near the membrane enhance the availability of reaction sites. The three-layered gradient design, particularly the L-M-S configuration, demonstrated superior oxygen distribution, reduced concentration gradients, and increased current density by 15.4%. These findings underline the importance of optimizing carbon sphere diameter distributions for enhancing CL performance. This study offers a novel framework for designing catalyst layers with improved mass transport and electrochemical efficiency, providing significant insights for the future development of high-performance PEMFCs.

## 1. Introduction

In recent years, the escalating global energy crisis and growing environmental concerns have driven strong interest in efficient and sustainable energy conversion technologies [1,2]. Among these, the proton exchange membrane fuel cell (PEMFC) has emerged as a leading green energy technology due to its high energy conversion efficiency, low operating temperature, rapid start-up capability, and minimal environmental footprint [3,4,5]. Nevertheless, the large-scale commercialization of PEMFCs remains constrained by the high cost and limited utilization efficiency of platinum catalysts [6], as well as by the complex coupling effects among mass transport and electrochemical processes within the catalyst layer (CL) [7]. To enhance catalyst utilization and optimize mass transport characteristics, extensive research has focused on the structural design and engineering of the CL [8,9,10].

Experimentally, numerous studies have achieved gradient regulation of catalyst loading, pore structure, and ionomer distribution using various fabrication methods, such as spraying [11], filtration [12], and doctor-blading [13]. Ye et al. fabricated a dual-layer CL with gradients in platinum content and pore size via filtration, achieving approximately 11% higher single-cell performance [14]. Shahgaldi et al. prepared a cathode catalyst layer (CCL) with an ionomer gradient by spraying, leading to a 13% performance improvement through enhanced proton and gas transport [15]. Ayoub et al. constructed a continuous ionomer gradient using a two-step doctor-blade process, which improved proton conduction and performance stability under varying humidity [16]. Chan et al. developed carbon nanofiber-based CLs with tunable structural gradients, demonstrating significant improvements in both conductivity and mass transport [17]. Chen et al. used ultrasonic spraying to create dual gradients in Pt/C ratio and Nafion content, improving low-humidity performance by up to 135.7% [18]. Although such experiments demonstrate clear benefits, they are time-consuming and costly, and establishing quantitative correlations between macroscopic structure and microscopic transport remains challenging. Consequently, numerical simulation has become an essential tool for investigating gradient-structured CLs.

Multiphysics models based on coupled multiphase transport mechanisms have been widely applied to analyze the influence of gradient parameters, such as catalyst loading, porosity, and ionomer distribution, on PEMFC performance [19,20]. Li et al. demonstrated that a dual-layer gradient porous CL with increasing pore size enhances phosphoric acid distribution and oxygen diffusion, markedly improving performance [21]. Roshandel et al. showed that increasing catalyst concentration in high-reaction-rate regions improves utilization and increases maximum power density by 6.8% [22]. Li et al. further found that a decreasing platinum loading profile along the flow channel enhances oxygen utilization and temperature uniformity [23]. Sabzpoushan et al. employed a two-dimensional, non-isothermal, two-phase model to show that increasing platinum loading along the flow direction improves oxygen supply and raises power density and voltage by 1.6% and 5%, respectively [24]. Wang et al. proposed a triple-gradient Nafion distribution that simultaneously optimized oxygen diffusion and proton conductivity, achieving a 6.8% power density increase [25]. Xuan et al. reported that rational gradient designs improve reaction uniformity and increase limiting current density by 64.5%, while also proposing a predictive model for optimal gradient structures [26]. These studies highlight the critical role of gradient structures in improving transport and electrochemical uniformity; however, traditional continuum models still struggle to capture fine-scale multiphase flow in complex porous media.

To resolve these limitations, the lattice Boltzmann method (LBM) has been increasingly adopted for pore-scale analysis of multiphase transport in CLs [27,28,29]. Benefiting from its strengths in modeling complex geometries, porous media, and interfacial dynamics, LBM provides deep insights into microscale diffusion, phase change, and reaction kinetics. Xu et al. applied pore-scale LBM to study heterogeneous reaction flow and ice-melting during PEMFC cold start, revealing that platinum content, ionomer fraction, and carbon support size strongly affect oxygen transport and melting rate [30]. Shen et al. developed a three-dimensional LBM model and found that increasing platinum loading near the membrane interface and along the flow direction improves platinum utilization and current uniformity, leading to a 0.83% performance gain [31]. Hou et al. employed a pore-scale model to demonstrate that higher Pt/C ratios and optimized ionomer-to-carbon (I/C) ratios reduce mass-transfer losses while maintaining large active areas, resulting in a 50% improvement in performance [32]. Collectively, these studies confirm that LBM effectively captures gas diffusion, liquid water formation, and phase transition phenomena, offering powerful guidance for rational CL design.

The particle size of carbon supports or platinum nanoparticles critically affects catalytic activity and transport behavior. Zhang et al. found that moderately increasing Pt particle size reduces mass-transfer resistance and improves catalyst utilization in ultrathin electrodes [33]. Xu et al. synthesized Pt/C catalysts with particle sizes from 2.9 to 6.5 nm and observed that larger particles decrease surface defects and oxide adsorption, enhancing specific activity and stability [34]. Yano et al. prepared monodisperse 2 nm Pt/C catalysts with excellent activity and durability during accelerated aging tests [35]. Zhang et al. incorporated carbon nanotubes and graphene oxide to tailor the pore structure of carbon supports, thereby reducing oxygen diffusion resistance and improving electrochemical durability [36]. Perez et al. showed that Pt/C catalysts with 2.6 nm particles exhibited the highest mass activity in ethanol oxidation, though this effect was less pronounced in full-cell operation due to intermediate-product interference [37]. Zheng et al. demonstrated that multilayer gradient Pt distributions mitigate platinum dissolution and optimize electrochemical surface area, enhancing durability and lifetime [38]. These studies collectively show that particle size influences both active surface area and reactant diffusion. However, the effects of through-plane particle size gradients remain largely unexplored.

In summary, previous experimental and numerical investigations have revealed the significance of gradient structures in enhancing mass transport and catalytic activity within PEMFC CLs. Yet, the role of carbon particle size distribution, especially through-plane gradient, on nanoscale transport remains insufficiently understood. Conventional models often neglect the coupled effects of pore morphology, oxygen diffusion pathways, and electrochemical kinetics. To address this gap, the present study employs a pore-scale LBM framework to systematically investigate how carbon sphere size gradients regulate oxygen transport, proton conduction, and electrochemical performance. The results provide mechanistic insights into the structure–performance relationship and offer theoretical guidance for the rational design of next-generation gradient-engineered electrodes.

## 2. Methodology

### 2.1. Microscopic Stochastic Reconstruction Method for Gradient CLs

To accurately reconstruct the three-dimensional microstructure of the CL, this study adopts a multi-step stochastic modeling approach. This method systematically simulates key processes including carbon support growth, platinum nanoparticle deposition, and ionomer coverage. The reconstructed model generated through this approach quantitatively captures the nanoscale spatial distribution of carbon supports, platinum particles, and ionomers within the CL, offering a novel framework for the quantitative characterization of CL microstructures.

The volume fractions of each component in the CL are determined by several key structural parameters, including platinum loading *γ_Pt_*, electrolyte content *θ_i_*, platinum-to-carbon mass ratio *θ_Pt/C_*, porosity *ε*, and catalyst layer thickness *δ*. By establishing mathematical relationships among these parameters and performing systematic derivations, the volume proportions can be precisely quantified. These results serve as a critical quantitative foundation for microstructural design and performance optimization of the CL. The specific methodology is detailed as follows [32].

(1) $$\varepsilon_{C}=(1-\varepsilon )\frac{1/\rho_{C}}{\theta_{Pt/C}/\rho_{Pt}+1/\rho_{C}+(1+\theta_{Pt/C})\theta_{i}/(\rho_{i}(1-\theta_{i}))}$$

(2) $$\varepsilon_{Pt}=(1-\varepsilon )\frac{\theta_{Pt/C}/\rho_{C}}{\theta_{Pt/C}/\rho_{Pt}+1/\rho_{C}+(1+\theta_{Pt/C})\theta_{i}/(\rho_{i}(1-\theta_{i}))}$$

(3) $$\varepsilon_{i}=(1-\varepsilon )\frac{\left(1+\theta_{Pt/C})\theta_{i}/(\rho_{i}(1-\theta_{i})\right)}{\theta_{Pt/C}/\rho_{Pt}+1/\rho_{C}+(1+\theta_{Pt/C})\theta_{i}/(\rho_{i}(1-\theta_{i}))}$$

(4) $$\gamma_{Pt}=\varepsilon_{Pt}\rho_{Pt}\delta$$

In the equations, *ε_C_*, *ε_Pt_*, and *ε_i_* denote the volume fractions of the carbon support, platinum particles, and ionomer, respectively. The corresponding material densities are defined as follows: carbon support density *ρ_C_* = 1800 mg/cm^3^, platinum density *ρ_Pt_* = 21,450 mg/cm^3^, and ionomer density *ρ_i_* = 2000 mg/cm^3^ [32]. In this study, the lattice resolution for numerical simulations is set to 1 lattice unit (lu) = 3 nm, providing a consistent spatial reference for the quantitative analysis of the catalyst layer microstructure. The baseline simulation parameters are as follows: platinum loading *γ_Pt_* = 0.3 mg/cm^2^, CL porosity *ε* = 0.35, platinum-to-carbon mass ratio *θ_Pt/C_* = 0.6, and ionomer content *θ_i_* = 1/3. Based on these parameter settings, a gradient design study of carbon particle diameters was conducted to investigate its impact on CL performance.

#### 2.1.1. Stochastic Reconstruction of Carbon Sphere Size Distributions

Based on the calculations from Equations (1)–(4) and referencing the baseline case reported by Hou et al. (*ε_C_* = 0.3465, 8–22 nm), carbon supports are assumed to be spherical for pore-scale reconstruction, and this study designs multiple cases with varying carbon sphere diameter ranges and through-plane layered distributions to evaluate the influence of carbon sphere size distribution on oxygen transport and electrochemical performance in the catalyst layer. The construction process of the carbon sphere network model is as follows: (1) the required number of carbon spheres, M, is estimated according to the target carbon volume fraction. Using a random function, the coordinates of sphere centers and their radii are generated, and the corresponding lattice nodes are assigned as the carbon phase. By introducing pseudo-random modulation of the local carbon sphere diameters, a non-uniform particle size distribution is achieved. (2) The actual carbon volume fraction of the generated model is then calculated. If the deviation from the target value exceeds 1%, the number of spheres M is adjusted and a new parameter set is regenerated. This generation–verification loop is iterated until the target precision is satisfied. Through these steps, precise control of the designed carbon sphere diameter distributions is ultimately realized.

#### 2.1.2. Deposition of Platinum Nanoparticles and Ionomer Coating

In this model, the size of each platinum nanoparticle is set to match the resolution of the lattice cells and adheres either to the surface of carbon supports or to existing Pt particles. The algorithm is implemented through the following steps: (1) All adjacent vacant lattice positions surrounding carbon spheres and existing Pt particles are systematically searched and recorded as potential Pt attachment sites. (2) A random subset equivalent to 10% of the total number of target Pt particles is selected from the identified potential sites. These selected points are then converted into Pt particles. This batch-based deposition strategy promotes a uniform spatial distribution. (3) Steps (1) and (2) are repeated until the desired number of Pt particles is reached. This algorithm maintains the stochastic nature of Pt particle distribution while enhancing spatial dispersion, thus more accurately replicating the microstructural characteristics of actual CLs.

The ionomer phase is distributed using a random probability strategy analogous to the platinum particle deposition algorithm, aiming to replicate its inherently non-uniform dispersion characteristics. By precisely controlling the deposition probability threshold, this method preserves the heterogeneous spatial distribution of the ionomer while effectively suppressing excessive local aggregation. As a result, it more accurately reflects the microscopic dispersion behavior of ionomer oligomers within the actual CL.

**Figure 1:**
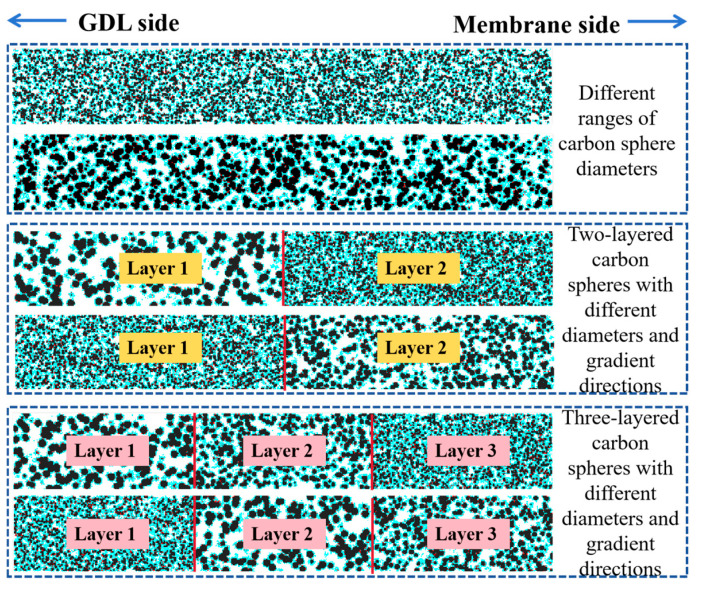
The 2D cross-section schematic diagrams of the 3D catalyst layer structure distributed along the thickness direction.

The schematic diagram in Figure 1 illustrates the catalyst layer structure, where the carbon spheres exhibit different diameter ranges and are distributed in two or three layers along the thickness direction. The black, blue, and red colors represent the carbon support, ionomer, and platinum, respectively. From a two-dimensional perspective, this effectively demonstrates the micro-random reconstruction method for the gradient catalyst layer, showing the successful creation of a non-uniform carbon sphere diameter distribution. It also ensures that the platinum particles and ionomer are discretely and reasonably distributed within the computational domain.

### 2.2. LB Model for Oxygen Transport and Electrochemical Reaction

The LBM integrates principles from molecular dynamics and statistical mechanics to establish a cross-scale computational framework that bridges macroscopic fluid behavior with microscopic particle dynamics. One of the key advantages of LBM is its superior capability to efficiently handle complex boundary conditions, making it particularly well-suited for accurately simulating flow fields within porous media characterized by irregular pore geometries. In CLs, where pore sizes are typically on the nanometer scale, oxygen transport predominantly occurs under low Reynolds number conditions, with diffusion serving as the primary mass transfer mechanism. Accordingly, the governing equations describing oxygen transport within the CL are given as follows [39]:

(5) $$\frac{\partial C_{O_{2}}}{\partial t}=\nabla \cdot \left(D_{O_{2}}\nabla C_{O_{2}}\right)+S_{O_{2}}$$

To simulate oxygen mass transport within the CL, this study adopts the D3Q7 LB model, which is based on seven discrete velocity directions. This model captures the transport behavior of species through a defined set of discrete velocities. The evolution of the oxygen concentration field is governed by the following LB evolution equation:

(6) $$g_{i}(x+c_{i}\Delta t,t+\Delta t)=g_{i}(x,t)-\omega [g_{i}(x,t)-g_{i}^{eq}(x,t)]+w_{i}S_{O_{2}}$$

Here, *ω* denotes the relaxation frequency, *c_i_* represents the discrete velocity vector in the *i*-th direction, and *w_i_* is the corresponding weight coefficient. The specific forms of the equilibrium distribution function *g^eq^* and the discrete velocity set *c_i_* are given as follows:

(7) $$g_{i}^{eq}=\frac{C}{7},i=0-6$$

(8) $$c_{i}=\left\{\begin{array}{l} (0,0,0),i=0\\ (\pm 1,0,0),(0,\pm 1,0),(0,0,\pm 1),i=1-6 \end{array}\right.$$

The oxygen concentration *C* is calculated as follows:

(9) $$C=\sum_{i=0}^{6}g_{i}$$

*S_O_*_2_ serves as a source of oxygen consumption. Due to substantial differences in reaction kinetics and local pore structures between active reaction sites and inactive sites, it is essential to distinguish between these two types of sites.

(10) $$S_{O_{2}}=\left\{\begin{array}{lll} -\frac{Hni}{RT\Delta L\cdot 4F}, & \mathrm{Active}\;\mathrm{site} & \\ 0, & \mathrm{Inactive}\;\mathrm{site} & \end{array}\right.$$

The electrochemical reaction system involves several key parameters, including the universal gas constant *R*, thermodynamic temperature *T*, lattice resolution Δ*L*, Faraday constant *F*, and the number *n* of adjacent active platinum sites. The parameter *n* directly influences the effective contact area and catalytic efficiency of the reaction. The electrochemical reaction rate *i* is calculated using the following equation:

(11) $$i=\frac{i_{ref}C_{O_{2},i}}{C_{O_{2},ref}}\exp\left(\frac{\alpha F\eta }{RT}\right)$$

In the equation, *i_ref_* denotes the reference current density, while *C_O_*_2_*_,i_* and *C_O_*_2_*_,ref_* represent the oxygen concentration within the ionomer phase and the reference oxygen concentration, respectively.

In electrochemical reactions, oxygen molecules undergo three consecutive steps:

(1) Dissolution into the ionomer phase;
(2) Diffusion and transport through the ionomer to the surface of platinum particles;
(3) Reaction with protons that migrate to the platinum surface via the ionomer network.

Because the gas-phase diffusion coefficient of oxygen in the CL pores is significantly higher than its diffusion coefficient in the ionomer phase, a pronounced concentration discontinuity exists at the ionomer/pore interface. The relationship between oxygen concentrations on both sides of this interface can be described by the following equation:

(12) $$\frac{RT}{H}=\frac{C_{O_{2},i}}{C_{O_{2},pore}}$$

(13) $$H=0.101325e^{\left(\frac{-666}{T}+14.1\right)}$$

In the above equation, *C_O_*_2_*_,pore_* denotes the oxygen concentration within the pore phase, and *H* is Henry’s constant, which is temperature-dependent.

Given the substantial difference in oxygen diffusion coefficients between the pore phase and the ionomer phase in the CL, it is crucial to accurately determine the diffusion coefficients in each medium. During oxygen transport within the pores, both bulk diffusion and Knudsen diffusion effects must be considered. The corresponding calculation equations are as follows:

(14) $$D_{\mathrm{bulk}}=\frac{2.2\times 10^{-5}{\left(\frac{T}{293.2}\right)}^{1.5}}{\left(\frac{P}{1.013\times 10^{5}}\right)}$$

(15) $$D_{Kn}=\frac{d_{p}}{3}{\left(\frac{8RT}{\pi M_{O_{2}}}\right)}^{0.5}$$

(16) $$D_{pore}={\left(\frac{1}{D_{bulk}}+\frac{1}{D_{Kn}}\right)}^{-1}$$

Here, *d_p_* represents the effective pore diameter. During calculation, the expansion proceeds along the direction of the pore lattice until a non-pore lattice is encountered. The effective pore diameter is then determined by averaging the distances in all directions. The diffusion coefficient of oxygen in the ionomer phase can be calculated using the following equation [40]:

(17) $$D_{i}=10^{-10}(0.1543(T-293)-1.65)$$

After determining the diffusion coefficients of oxygen in both the pore phase and the ionomer phase, the relaxation frequency *ω* in Equation (6) can be calculated as follows:

(18) $$\omega ={\left(3D+0.5\right)}^{-1}$$

### 2.3. Computational Domain and Boundary Conditions

The size of the simulation domain is set to 150 × 150 × 1069 lu^3^, corresponding to a physical scale of 450 × 450 × 3207 μm^3^. The boundary conditions are defined as follows:

(1) Surfaces of carbon supports and platinum particles: A half-way bounce-back scheme is applied;
(2) Lateral boundaries parallel to the Z-axis: Periodic boundary conditions are imposed.

The first lattice layer at z = 1 lu, located at the bottom of the Z-direction, is defined as the gas inlet. This plane corresponds to the gas diffusion layer (GDL) and CL interface and is assigned a constant inlet oxygen concentration, *C_O_*_2,_*_in_*. The unknown distribution function g6 at the inlet is then calculated as follows:

(19) $$g_{6}=(w_{5}+w_{6})C_{O_{2},in}-g_{5}$$

The last lattice layer in the Z-direction, located at z = 1069 lu, is designated as the gas outlet. This plane corresponds to the cross-section of the CL. By applying open boundary conditions at this location, the unknown distribution function *g*_5_ at the outlet can be calculated accordingly.

(20) $$g_{5}(x,y,1069)=g_{5}(x,y,1068)$$

To accurately simulate the concentration discontinuity of oxygen at the ionomer/pore interface, the boundary conditions in this interfacial region are defined as follows:

(21) $$g_{i}(x_{p},t+\Delta t)=\frac{1}{-\frac{1}{4k_{dis}}\frac{\Delta x}{\Delta t}-\frac{1}{H}-1}\left[\frac{1}{-\frac{1}{4k_{dis}}\frac{\Delta x}{\Delta t}+\frac{1}{H}-1}{\hat{g}}_{i}(x_{p},t+\Delta t)-2{\hat{g}}_{i}(x_{i},t+\Delta t)\right]$$

(22) $$g_{i}(x_{p},t+\Delta t)=g_{i}(x_{i},t+\Delta t)=\frac{1}{-\frac{1}{4k_{dis}}\frac{\Delta x}{\Delta t}-\frac{1}{H}-1}\left[\frac{1}{-\frac{1}{4k_{dis}}\frac{\Delta x}{\Delta t}-\frac{1}{H}+1}{\hat{g}}_{i}(x_{i},t+\Delta t)-\frac{2}{H}{\hat{g}}_{i}(x_{p},t+\Delta t)\right]$$

Here, *x_p_* and *x_i_* correspond to the pore node and ionomer node at the ionomer/pore interface, respectively. *g_i_* denotes the post-collision distribution function, and $\bar{i}$ represents the distribution function or velocity direction opposite to direction *i*.

In this study, key physical conversion factors for the LB model are defined as follows:

Length conversion factor (lattice resolution): Δ*l* = 3 × 10^−9^ m; Concentration conversion factor: Δ*C* = 1 mol/m^3^; Diffusion coefficient conversion factor: To ensure model stability, the minimum oxygen diffusion coefficient in the ionomer phase is used, Δ*D* = 5.7413 × 10^−8^ m^2^/s; Time conversion factor: Derived from Δ*l* and Δ*D*, calculated as Δ*t* = 1.5676 × 10^−10^ s; Source term conversion factor: Based on Δ*C* and Δ*t*, calculated as Δ*S* = 6.3793 × 10^9^ mol/(m^3^·s).

## 3. Results and Discussion

To elucidate the influence of carbon sphere diameter variation on the CL performance in PEMFCs, a series of lattice Boltzmann method simulations were conducted across three representative structural configurations. These include different ranges of carbon sphere diameters, two-layered CLs with segmented diameter variations, and three-layered CLs with gradient structures along the thickness direction. For each case, the pore size distribution (PSD), electrochemically active surface area (ECSA), oxygen concentration profiles, oxygen consumption rates, and resulting current densities were systematically analyzed. The outcomes provide critical insights into the structure–function relationship of CL morphology, highlighting the role of spatial heterogeneity in optimizing transport and reaction characteristics within PEMFCs.

### 3.1. Effect of Carbon Sphere Diameter Range on CL Transport and Reaction Characteristics

A total of 6 CL structures with varying carbon sphere diameters were investigated to evaluate the impact of uniform particle size and size ranges on CL performance. The structural characteristics of these cases are summarized in Table 1, where *R_c_min_* and *R_c_max_* represent the minimum and maximum carbon sphere radius, respectively. All CLs were constructed with identical total thicknesses of 3207 nm, and exhibit comparable bulk porosity, ranging from 0.3475 to 0.3595, carbon volume fractions (*ε_C_*), and high connectivity rates exceeding 99.69%, indicating well-connected carbon networks across all cases. The ECSA varies slightly, with a maximum deviation of approximately 14.5% across the cases, primarily due to differences in particle size and packing effects. These similarities in porosity, carbon content, and connectivity ensure that the observed differences in mass transport and electrochemical performance can be attributed primarily to the variation in carbon sphere diameters rather than structural randomness, thus providing a reliable basis for the comparative analysis in subsequent sections.

**Figure 2:**
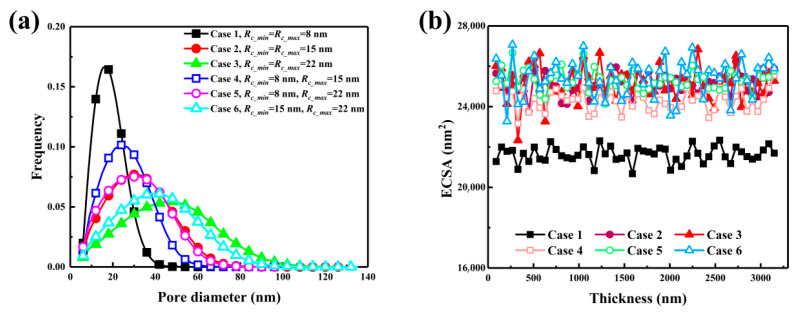
Effect of carbon sphere diameter variation on (**a**) PSD and (**b**) ECSA along the thickness direction of CLs.

Figure 2 illustrates the pore size distributions and through-plane variations in electrochemically active surface area for six catalyst layer configurations with different carbon sphere diameters, aiming to elucidate the effect of particle size on CL microstructure and reactive surface properties. As shown in Figure 2a, the pore size distribution (PSD) is highly dependent on the carbon sphere diameter. Case 1, composed entirely of 8 nm carbon spheres, exhibits a narrow PSD centered around 20 nm with a peak frequency of 0.165, indicating a densely packed structure with limited porosity. By contrast, Case 3 shifts the PSD peak to 45 nm and displays an extended tail beyond 100 nm, reflecting coarser packing and enlarged pores. The baseline configuration demonstrates a balanced PSD with both fine and coarse pores. Cases 4 and 6 show broadened PSDs; notably, Case 6 exhibits a distinct peak between 40 and 60 nm and a long tail, implying increased pore volume and enhanced multiscale connectivity, which favors oxygen diffusion.

Figure 2b shows the ECSA distribution across the CL thickness. Case 1 yields the lowest average ECSA (21,611 nm^2^) due to limited surface exposure and compact packing. In contrast, configurations containing larger or polydisperse carbon spheres (Cases 2, 3, 5, 6) achieve markedly higher average ECSAs exceeding 25,000 nm^2^. Case 6 reaches the maximum value (25,346 nm^2^), demonstrating that combining mid-sized and large particles effectively increases the reactive surface area while maintaining good structural connectivity. Moreover, these configurations exhibit relatively uniform ECSA profiles across the through-plane direction, suggesting consistent catalyst accessibility. These findings confirm that the carbon sphere size distribution critically governs pore architecture and electrochemical activity. Properly engineered particle arrangements can promote hierarchical porosity and simultaneously enhance the available ECSA.

**Figure 3:**
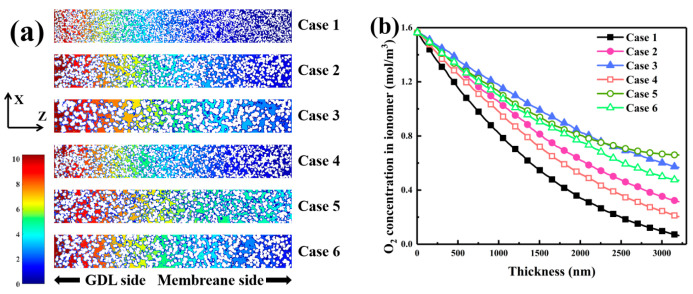
Oxygen concentration characteristics in CLs with different carbon sphere diameter distributions. (**a**) Oxygen concentration distribution at cross-section Y = 75. (**b**) Oxygen concentration profiles in the ionomer phase along the CL thickness.

Figure 3 presents oxygen concentration profiles within the CL under different carbon sphere size distributions, providing insight into the impact of particle size on oxygen transport. As shown in Figure 3a, significant variations occur in the oxygen concentration field at the Y = 75 nm cross-section. In Case 1, the concentration remains relatively uniform, peaking at 1.6 mol/m^3^, indicative of a compact structure that restricts diffusion but yields smooth gradients. Conversely, Case 3 exhibits higher oxygen concentrations near the membrane interface, implying that larger carbon spheres improve pore connectivity and facilitate deeper oxygen penetration. Cases 5 and 6 display similarly smoother gradients and broader distributions, reflecting more effective transport pathways produced by mixed particle sizes.

Figure 3b depicts the oxygen concentration profiles along the through-plane direction. In Case 1, the concentration declines sharply from 1.6 mol/m^3^ at the GDL side to nearly zero at 3000 nm, signifying diffusion limitation caused by tightly packed small pores. Case 3 shows a slower decay, maintaining 1.0 mol/m^3^ at 1500 nm and 0.55 mol/m^3^ at 3000 nm, indicating improved multiscale connectivity and enhanced oxygen diffusion. Case 5, containing 8 nm and 22 nm spheres, also exhibits a more gradual decline, delivering better oxygen availability than the single-size case. Overall, larger carbon spheres substantially enhance pore interconnectivity and oxygen diffusion, while smaller spheres restrict deep transport. A mixed particle-size distribution provides an optimal balance between pore continuity and diffusive efficiency, promoting higher catalytic reaction rates within the CL.

**Figure 4:**
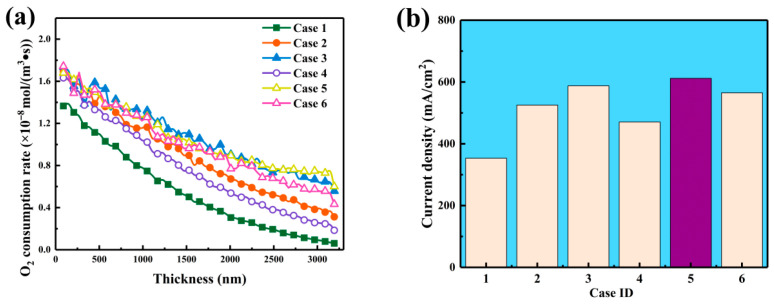
Effects of carbon sphere diameter distribution on oxygen consumption rate and current density. (**a**) Oxygen consumption rate profiles along the CL thickness direction. (**b**) Resulting current densities for the 6 CLs.

Figure 4 demonstrates the influence of carbon sphere diameter range on oxygen consumption rate and current density. In Figure 4a, the oxygen consumption rate across the CL thickness reflects the influence of pore morphology. For Case 1, the rate starts at 1.37 mol·m^−3^·s^−1^ and rapidly drops to near zero at 3000 nm, confirming diffusion constraints imposed by small particle sizes. Case 3 shows a slower decline, from 1.49 mol·m^−3^·s^−1^ at the surface to 0.92 mol·m^−3^·s^−1^ at 3000 nm, demonstrating that larger spheres enhance multiscale connectivity and sustain higher oxygen utilization in deeper regions. Case 5 exhibits a similar trend, maintaining 0.85 mol·m^−3^·s^−1^ at 3000 nm, indicating that a mixed particle-size configuration preserves oxygen supply throughout the CL.

Figure 4b compares the resulting current densities for different CL structures. Case 1 produces the lowest current density of 353.49 mA·cm^−2^, confirming that small carbon spheres hinder pore connectivity and charge transport. Increasing the carbon sphere size improves both pore continuity and electron conduction: the current density rises to 525.24 mA·cm^−2^ for Case 2 and 587.73 mA·cm^−2^ for Case 3. Case 4 achieves 470.48 mA·cm^−2^, higher than Case 1 but below the large-sphere cases, suggesting limited improvement from partial mixing. Case 5 exhibits the highest current density of 611.88 mA·cm^−2^, demonstrating that combining medium and large carbon spheres yields the most favorable balance of pore structure, oxygen transport, and electrical performance. Overall, increasing carbon sphere diameter enhances pore interconnectivity and oxygen accessibility, while mixed-size distributions further optimize transport and electrochemical efficiency within the catalyst layer.

### 3.2. Effect of Two-Layered Carbon Sphere Diameter Distribution on Catalyst Layer Characteristics

Building on the findings in Section 3.1, Section 3.2 investigates a two-layered carbon sphere diameter distribution in the catalyst layer to determine whether distinct diameter regions can enhance performance by improving mass transport and active site utilization. Six configurations were developed with different carbon sphere diameters in two segments, as shown in Table 2. The segment near the gas diffusion layer is denoted *R*_1_, and that adjacent to the membrane is *R*_2_. All catalyst layers had a constant thickness of 3207 nm, ensuring that observed variations resulted solely from particle size differences. To isolate orientation effects, Case 8 was the reverse of Case 7, and Cases 10 and 12 were reversed versions of Cases 9 and 11. All structures exhibited high connectivity between 99.50% and 99.68%, indicating excellent pore continuity. Case 11 achieved the highest electrochemically active surface area of 2.71 × 10^7^ nm^2^ when *R*_1_ was 15 nm and *R*_2_ was 22 nm, demonstrating enhanced reactivity through improved porosity and surface exposure.

**Figure 5:**
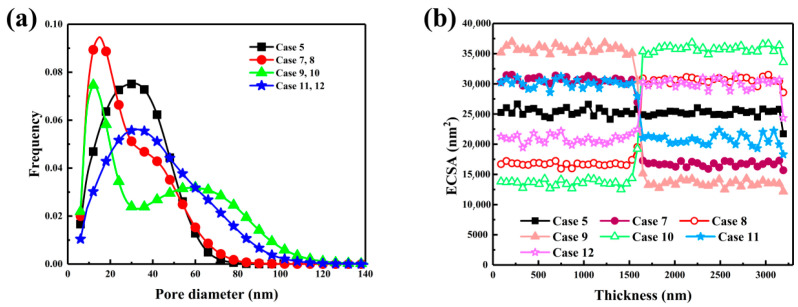
Effects of two-layered carbon sphere diameter distribution on catalyst layer structural characteristics. (**a**) Pore size distribution and (**b**) ECSA variation.

Figure 5a shows that the two-layer configuration strongly alters the pore size distribution. When the diameter difference was large, as in Case 9 with *R*_1_ of 8 nm and *R*_2_ of 22 nm, the distribution exhibited two peaks, one at 20–40 nm with a frequency of 0.089 and another extending to 60–100 nm. This bimodal structure arose from small spheres forming mesoporous networks near the gas diffusion layer and large spheres creating macropores near the membrane. In contrast, Case 7, with *R*_1_ of 8 nm and *R*_2_ of 15 nm, showed a single peak at 30–50 nm with a frequency of 0.074, indicating limited pore diversity. Case 11, with *R*_1_ of 15 nm and *R*_2_ of 22 nm, displayed a broad distribution centered between 40 and 60 nm with a frequency above 0.03, showing improved meso-macro connectivity and low-resistance oxygen pathways. Figure 5b illustrates the influence of the gradient on the through-plane ECSA. When large particles were placed near the membrane, as in Case 10, ECSA rose across the layer and reached 25,346 nm^2^ at mid-depth, much higher than the homogeneous layer’s 21,611 nm^2^. This resulted from the open packing of large particles that increased Pt/C exposure, while smaller particles on the gas diffusion layer side preserved surface area. Reversing the gradient, as in Case 9, reduced ECSA below 22,000 nm^2^ due to dense packing of small particles. Large particles near the membrane improved proton transport and triple-phase interface utilization, while small particles near the gas diffusion layer enhanced oxygen diffusion. However, excessive densification limited site accessibility. Case 11, combining large particles near the membrane with medium ones near the gas diffusion layer, achieved balanced optimization and improved oxygen uniformity.

**Figure 6:**
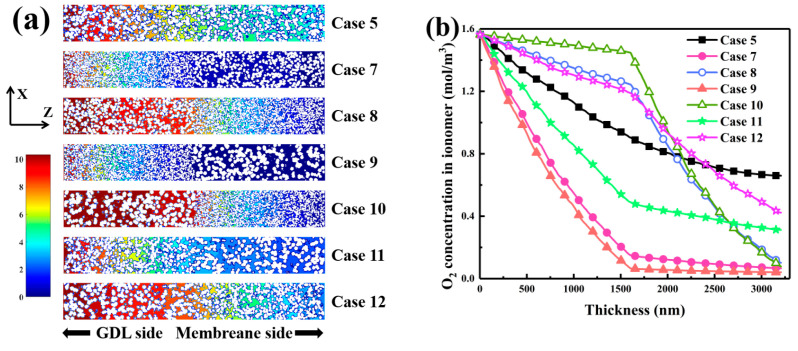
Oxygen concentration characteristics in two-layered CLs with gradient of carbon sphere diameter. (**a**) Oxygen concentration distribution at cross-section Y = 75. (**b**) Oxygen concentration profiles in the ionomer phase along the CL thickness.

Figure 6a reveals that the two-layered gradient substantially affects oxygen distribution. When large particles were located on the membrane side, continuous low-tortuosity channels formed with smooth isoconcentration contours, suppressing oxygen-depleted regions. The small-particle zone near the gas diffusion layer provided a dense network of micropores with high surface area, yielding synergistic transport and reaction behavior. When the gradient was reversed and small particles occupied the membrane side, steep oxygen gradients appeared, forming depletion zones near the membrane. This indicates that a controlled particle-size gradient couples structure and function to optimize performance. Figure 6b quantifies oxygen concentration along the thickness. The initial value at the gas diffusion layer inlet was 1.56 mol·m^−3^ for all cases. When large particles were placed near the membrane, as in Case 10, oxygen concentration remained 1.46 mol·m^−3^ at mid-depth, compared with 0.94 mol·m^−3^ in the homogeneous layer, and decreased gradually to 0.17 mol·m^−3^ at 3000 nm. The reversed gradient in Case 9 caused a sharp drop to 0.04 mol·m^−3^ at 3000 nm due to restricted diffusion. The intermediate Case 11 provided a balanced profile with 1.26 mol·m^−3^ at 1500 nm and 0.19 mol·m^−3^ at 3000 nm. These results show that combining medium and large particles optimizes diffusion and reaction balance. For thin layers, placing large particles near the membrane and smaller ones near the gas diffusion layer enhances oxygen supply, site utilization, and structural stability without increasing thickness.

**Figure 7:**
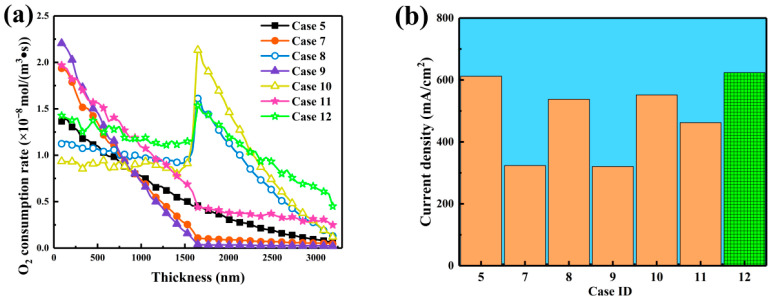
Effects of two-layered carbon sphere diameter gradient distribution on catalyst layer performance. (**a**) Oxygen consumption rate distribution along the CL thickness direction. (**b**) Current densities for the different CL configurations.

Figure 7a presents oxygen consumption rates across the thickness. All configurations showed high reaction intensity near the gas diffusion layer, decreasing with depth due to oxygen depletion. Cases 8, 10, and 12 exhibited peaks around 1500 nm with rates of 1.61, 2.13, and 1.54 mol·m^−3^·s^−1^, respectively, higher than those in Cases 7, 9, and 11. Placing large particles near the gas diffusion layer created high-permeability channels, delayed the reaction front, and maintained stronger frontal activity, alleviating oxygen starvation caused by dense structures. Figure 7b summarizes steady-state current densities. Case 12 achieved the highest value of 623.64 mA·cm^−2^, a 1.9% improvement over the homogeneous reference and much higher than its reversed configuration, Case 11, which produced 462.13 mA·cm^−2^. Case 10 reached 551.67 mA·cm^−2^, exceeding Case 9, which yielded 320.31 mA·cm^−2^. These results demonstrate that positioning large particles near the gas diffusion layer and smaller or medium ones near the membrane aligns the reaction zone with efficient transport paths. At the mesoscopic scale, this structure forms low-tortuosity macroporous channels that improve gas diffusion and delay the reaction front, while smaller particles near the membrane provide dense active sites. The direction of the carbon sphere diameter gradient is therefore a key factor coupling mass transport with electrochemical reactions, ultimately governing catalyst layer performance.

### 3.3. Effect of Three-Layered Carbon Sphere Diameter Distribution on Catalyst Layer Characteristics

In this section, six configurations of carbon sphere diameters, *R*_1_, *R*_2_, and *R*_3_, distributed across three catalyst layer regions-near the GDL, the middle section, and the membrane side were investigated. Structural parameters for these configurations are summarized in Table 3 to highlight the effect of three-layer carbon sphere diameter distribution on catalyst layer performance. The symbols S, M, and L in the Case ID column represent configurations with small, medium, and large local carbon sphere diameters, respectively. Although electrochemically active surface areas varied only slightly between 26,569,287 and 26,584,542 nm^2^, it was inferred that porosity and connectivity, rather than total surface area, exerted dominant influence on overall performance. By optimizing the diameter gradient, mass transport and reaction kinetics were enhanced as oxygen diffusion and active site accessibility were improved. Connectivity in all configurations remained between 99.52% and 99.53%, confirming excellent structural integrity that is essential for efficient mass transport. Minimal variation in porosity and carbon volume fraction suggested that the performance differences were primarily governed by the particle-size distribution rather than changes in macrostructure.

**Figure 8:**
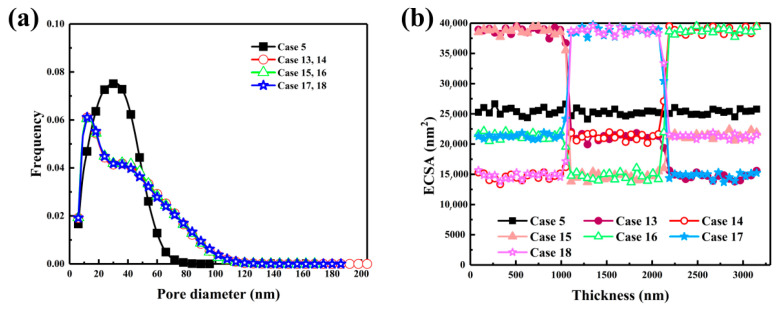
Effects of three-layered carbon sphere diameter distribution on catalyst layer structural characteristics. (**a**) Pore size distribution and (**b**) ECSA variation.

In Figure 8a, the regulation of pore size distribution by the three-layer diameter gradient is demonstrated. A distinct bimodal pattern was observed under the large-medium-small sequence, with a major peak between 20 and 40 nm and a secondary one extending to 100 nm. Low-tortuosity channels were formed by large spheres near the gas diffusion layer, while mesoporous regions below 50 nm were generated by small spheres on the membrane side. This hierarchical arrangement was found to enhance both oxygen transport and capillary infiltration. In the S-L-M sequence, a broader plateau appeared between 40 and 60 nm, indicating that the medium layer effectively smoothed abrupt diameter transitions and strengthened pore connectivity. In contrast, the M-S-L configuration yielded a narrower distribution, as densification induced by small mid-layer particles could not be compensated by large particles near the membrane. Figure 8b presents the through-plane variation in electrochemically active surface area. In the L-M-S configuration, ECSA was found to increase from the gas diffusion layer toward the membrane, peaking at 25,346 nm^2^ at 1500 nm, approximately 17% higher than that of the homogeneous structure. The enhancement was attributed to structural support provided by large spheres that delayed site blockage in the middle layer, while small spheres near the membrane maintained local surface area before a final decline occurred due to proton transport resistance. In the S-L-M configuration, ECSA rapidly decreased from 20,000 to 5000 nm^2^ as access to active sites was hindered near the gas diffusion layer. The highest and most stable ECSA was maintained in the M-L-S configuration, remaining above 22,000 nm^2^ between 2000 and 2500 nm. A balanced distribution of medium spheres at the front was shown to mitigate densification and extend proton penetration, increasing active site utilization by nearly 20%.

**Figure 9:**
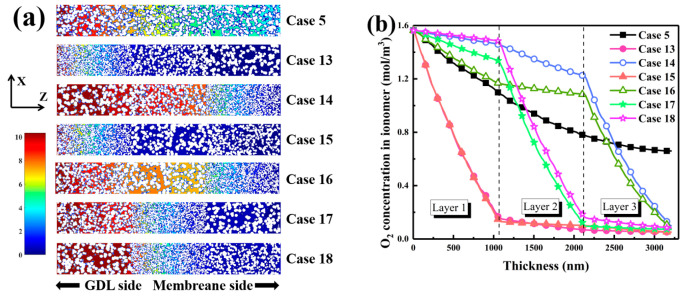
Oxygen concentration characteristics in three-layered CLs with gradient of carbon sphere diameter. (**a**) Oxygen concentration distribution at cross-section Y = 75. (**b**) Oxygen concentration profiles in the ionomer phase along the CL thickness.

In Figure 9a, oxygen concentration regulation by the three-layer gradient is revealed. Under the L-M-S arrangement, oxygen was transported evenly through low-tortuosity pathways, suppressing local depletion and maintaining equilibrium between consumption and replenishment. When small spheres were positioned near the gas diffusion layer, oxygen penetration was severely hindered, and low-concentration regions were observed near the inlet. Consequently, pore connectivity and tortuosity were determined by the gradient, which dictated the spatial alignment between transport and reaction zones.

Oxygen concentration profiles in the ionomer phase are quantified in Figure 9b. In the homogeneous reference, oxygen concentration gradually declined to 0.66 mol·m^−3^ near the membrane. In the L-M-S configuration, oxygen concentration decreased sharply to 0.14 mol·m^−3^ at 1000 nm, whereas in the S-L-M sequence, it dropped below 0.12 mol·m^−3^ at the same depth. When large particles were arranged near the gas diffusion layer and transitioned to small ones near the membrane, concentrations of 1.15 mol·m^−3^ at 2000 nm and 0.95 mol·m^−3^ at 2500 nm were maintained, indicating reduced resistance and delayed decay. In contrast, the M-S-L configuration exhibited only 0.39 mol·m^−3^ at 1500 nm, signifying a transport bottleneck caused by small mid-layer particles. It was therefore concluded that large particles near the gas diffusion layer constructed primary diffusion highways, medium particles buffered pore-size transitions, and small particles near the membrane provided dense active sites. A progressive L-M-S gradient was demonstrated to ensure sufficient oxygen supply in deeper regions and efficient catalyst utilization.

**Figure 10:**
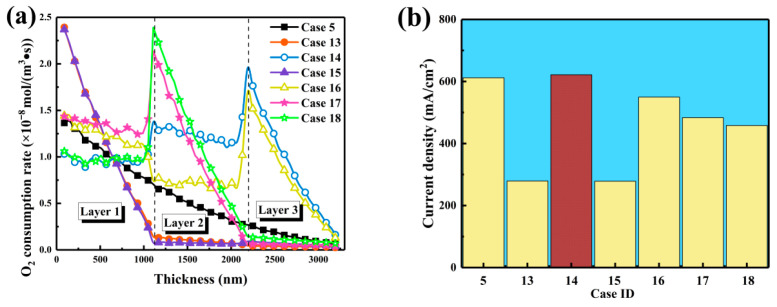
Effects of three-layered carbon sphere diameter gradient distribution on catalyst layer performance. (**a**) Oxygen consumption rate distribution along the CL thickness direction. (**b**) Current densities for the different CL configurations.

As shown in Figure 10a, oxygen consumption dynamics were strongly influenced by the diameter gradient. When small particles occupied the front, the reaction was concentrated at the inlet, with a maximum rate of approximately 2.4 × 10^−8^ mol·m^−3^·s^−1^, leading to rapid oxygen depletion and shallow reaction zones. When large particles were placed near the gas diffusion layer, the reaction front shifted deeper, and peaks near 2200 nm reached about 1.9 × 10^−8^ mol·m^−3^·s^−1^. This shift indicated that low-tortuosity channels allowed deeper oxygen penetration and improved balance between consumption and replenishment. When medium particles were located at the front, the reaction peak appeared around 1100 nm and decayed rapidly thereafter, indicating partial improvement yet remaining diffusion limitations. In Figure 10b, steady-state current densities corresponding to different gradients are presented. The L-M-S configuration achieved the highest current density of 621.68 mA·cm^−2^, slightly exceeding the homogeneous structure at 611.88 mA·cm^−2^. Enhanced performance was attributed to the formation of low-tortuosity diffusion channels by large particles near the gas diffusion layer and the provision of abundant active sites by small particles near the membrane. Configurations containing small mid-layers yielded lower outputs around 480 mA·cm^−2^, while S-L-M designs exhibited the lowest value near 279 mA·cm^−2^, consistent with pronounced inlet-side oxygen depletion. Mechanistically, it was demonstrated that placing large particles near the gas diffusion layer reduced tortuosity, enlarged effective pore sizes, and increased through-plane diffusion, enabling oxygen to penetrate deeper into the catalyst layer. The L-M-S gradient was therefore identified as the optimal configuration, providing wide diffusion channels in the front, structural buffering in the middle, and high active-site density near the membrane. This design stabilized the reaction front, ensured spatial matching between oxygen supply and consumption, and resulted in improved current density and overall catalyst layer performance.

## 4. Conclusions

In this study, the influence of non-uniform carbon sphere diameter distributions on the structural and electrochemical performance of catalyst layers in proton exchange membrane fuel cells was investigated using the lattice Boltzmann method. By analyzing three representative distribution patterns, it was demonstrated that tailored structural gradients markedly enhance oxygen transport and reaction kinetics within the catalyst layer.

The results reveal that carbon sphere size distribution critically governs pore structure and electrochemically active surface area. Gradient designs in both two-layer and three-layer configurations form hierarchical pore networks, improving multi-scale connectivity and facilitating efficient oxygen diffusion. Large spheres near the gas diffusion layer reduce tortuosity and strengthen oxygen transport, while small spheres near the membrane increase accessible reaction sites, thereby enhancing catalytic efficiency.

The optimal three-layer configuration, particularly the L-M-S sequence, yielded superior oxygen distribution, higher consumption rates, and greater current density, leading to more uniform reactions through the thickness. These findings highlight gradient design as an effective strategy for optimizing catalyst-layer microstructures and advancing high-performance, durable fuel cell systems.

## Figures and Tables

**Table 1:** Structural parameters of catalyst layers with varying carbon sphere diameters.

Case ID	*R_c_min_* (nm)	*R_c_max_* (nm)	CL Thickness (nm)	Bulk Porosity	*ε_C_*	Connected Rate	ECSA (nm^2^)
1	8	8	3207	0.3518	0.3450	99.74%	23,099,139
2	15	15	3207	0.3591	0.3379	99.70%	26,812,692
3	22	22	3207	0.3510	0.3462	99.72%	26,836,245
4	8	15	3207	0.3573	0.3396	99.70%	25,914,024
5	8	22	3207	0.3475	0.3465	99.69%	27,017,991
6	15	22	3207	0.3595	0.3376	99.72%	27,089,154

**Table 2:** Catalyst Layer Structure Information for Two-Layered Carbon Sphere Diameter Distribution.

Case ID	*R*_1_ (nm)	*R*_2_ (nm)	CL Thickness (nm)	Bulk Porosity	*ε_C_*	Connected Rate	ECSA (nm^2^)
7	8	15	3207	0.3523	0.3445	99.62%	25,301,898
8	15	8	3207	0.3523	0.3445	99.62%	25,301,898
9	8	22	3207	0.3529	0.3439	99.50%	26,376,057
10	22	8	3207	0.3529	0.3439	99.50%	26,376,057
11	15	22	3207	0.3581	0.3389	99.68%	27,144,009
12	22	15	3207	0.3581	0.3389	99.68%	27,144,009

**Table 3:** Catalyst Layer Structure Information for Three-Layered Carbon Sphere Diameter Distribution.

Case ID	*R*_1_ (nm)	*R*_2_ (nm)	*R*_3_ (nm)	Bulk Porosity	*ε_C_*	Connected Rate	ECSA (nm^2^)
13 (S-M-L)	8	15	22	0.3530	0.3439	99.52%	26,584,542
14 (L-M-S)	22	15	8				
15 (S-L-M)	8	22	15	0.3522	0.3447	99.52%	26,569,287
16 (M-L-S)	15	22	8				
17 (M-S-L)	15	8	22	0.3529	0.3440	99.53%	26,591,850
18 (L-M-S)	22	15	8				

## Data Availability

The original contributions presented in this study are included in the article. Further inquiries can be directed to the corresponding authors.
